# Encouraging reusability of computational research through Data-to-Knowledge Packages - A hydrological use case

**DOI:** 10.12688/openreseurope.20221.1

**Published:** 2025-05-06

**Authors:** Markus Konkol, Astra Labuce, Sami Domisch, Merret Buurman, Vanessa Bremerich

**Affiliations:** 1 52°North Spatial Information Research, Münster, 48155, Germany; 2 Institute of Aquatic Ecology, Agency of Daugavpils University, Riga, LV-1007, Latvia; 3 Forschungsverbund Berlin e.V., Leibniz Institute of Freshwater Ecology and Inland Fisheries (IGB), Berlin, 12587, Germany

**Keywords:** FAIR Data, Reproducible Research, Workflows, OGC API, Galaxy, Hydrology, Gulf of Riga, Trend analysis

## Abstract

The growing demand for reproducible research is based on the expectation that publishing research in this form will enable its reuse and the generation of new knowledge. However, reproducibility alone does not guarantee these benefits. Users still need to make considerable efforts to understand the data and analysis code before they can reuse these components in other contexts. To address this challenge, we introduce the Data-to-Knowledge Package (D2K-Package), a collection of research materials including source code and open FAIR data, virtual labs, web API services, and computational workflows. The D2K-Package’s core is the reproducible basis composed of the data and source code on which an analysis is based. This core is designed such that the other components can be derived from it. The main goal of the package is to help researchers generate new knowledge by facilitating the understanding and encouraging the reuse of reproducible research. We demonstrate the applicability of the D2K-Package with a hydrological use case which can be also used for testing, and discuss its seamless integration into the research cycle.

## Plain language summary

Researchers often collect data and analyse it using appropriate methods in order to answer a specific research question. The research results are then published in scientific articles. If other researchers have access to the data and the analysis used and can achieve the same results as in the article, this is called “reproducible research”. One of the main advantages of reproducible research is that other scientists can continue the work and reuse the materials. However, publishing the materials in a reproducible manner is a challenge for the developer of the analysis. In addition, reusing the material for others is a difficult task as the analysis can become very complex.

In this paper, we introduce the Data-to-Knowledge package (D2K-Package), which contains the data and analysis in a reproducible manner, as well as other components that help other researchers understand and reuse the analysis. One of these components is a workflow that visualizes the steps of the analysis pipeline as a first entry point. To demonstrate the feasibility of the D2K-Package, we applied the idea to a real use case in hydrology.

## Introduction

The scientific community must adapt to an environment in which research budgets are shrinking in many parts of the world, even though scientific advances are urgently needed to tackle global challenges such as climate change, biodiversity loss, and pandemics ^1, 2^. Sharing and publishing reusable research outputs is a crucial strategy to mitigate these challenges and foster new collaborative developments ^3^. A key concept is computational reproducibility, which ensures that other researchers can validate and verify the results reported in a scientific article using the same data and code ^4, 5^. Consequently, reproducibility is a fundamental quality criterion in science, and one might assume it is widely practiced to strengthen the credibility and transparency of research. However, several studies have shown that many research articles across disciplines are in fact not reproducible ^6– 8^ - a phenomenon often referred to as the "reproducibility crisis" ^9^. There are various reasons for this, but a major obstacle is the perception that publishing reproducible research results is time-consuming and not worth the investment ^10^. Nevertheless, reproducible research should be valued not only for its intrinsic scientific merit, including verification and validation, but also for its wider benefits. A key expectation is that reproducible research results are inherently more reusable by other researchers, reducing redundant efforts and accelerating the creation of new knowledge ^11– 13^.

In response, funding agencies, peer reviewers, and academic journals are increasingly emphasizing the need for researchers to publish all materials required for reproducibility ^14– 16^. While this emphasis represents a step forward, it also entails changes in researchers’ daily workflows, and this transition is still ongoing. Numerous tools, guidelines, and best practices have been developed to support this trend, including the FAIR (Findability, Accessibility, Interoperability, and Reusability) principles for data ^17^ and software ^18^ and the "Ten Simple Rules for Computational Reproducible Research” ^19^. Gentleman and Lang ^20^ introduced the concept of a compendium as a package including a dynamic document (i.e., the paper) and the code and data needed to produce the computational results in that document. ReproZip ^21^ is an example implementation of the compendium concept and packages the computational environment with the source code and data so that users can start a virtual machine to re-run the analysis. The commercial publisher CodeOcean creates such virtual environments as supplements for research articles ^22^. In addition, eLife is a scientific publisher that offers interactive papers based on a reproducible analysis ^23^.

Encapsulating all materials in a compendium might limit the findability of the individual components contained therein, unless the metadata of the individual elements can be found externally. Moreover, researchers may want or need to publish individual research assets in dedicated repositories. As an alternative to a compendium, a so-called Research Object can be created, which aggregates digital assets that are part of an analysis and are published in different repositories or archives ^24, 25^. Together with the Research Object, a particularly influential concept in the context of this paper is the Knowledge Package developed by the Group on Earth Observation (GEO), a “reusable application-sharing unit” that integrates geospatial datasets, code, tools, and the computational environment ^26^.

However, reusing research code requires a deep understanding of the underlying logic of the analysis pipeline, e.g., how the functions work, the meaning of input parameters, and how to adapt them to other contexts. Understanding complex source code requires knowledge of the programming language and can be a challenge even for experienced programmers ^27^. Consequently, researchers often tend to reinvent the wheel and develop analyses from scratch, or rely on proprietary, point-and-click software with a graphical user interface (GUI) that lacks reproducibility ^28^. This challenge is further complicated by the diverse needs and skills of different stakeholders, including reviewers, researchers, and science journalists. Workflow management systems can help with this problem by providing an entry point into the analysis pipeline. Examples include Nextflow ^29^, a command line-based tool that allows users to script workflows, and Snakemake ^30^, which defines workflow steps as rules. The Galaxy platform ^31^ also addresses users without programming expertise by offering an intuitive user interface for the development and execution of readily-sharable workflows.

In this paper, we introduce the Data-to-Knowledge Package (D2K-Package), a collection of links to digital research assets - such as data, code, virtual labs, web API services and workflows - that unlock the potential of open reproducible research and open FAIR data. Conceptually, the D2K-Package is built on top of the Research Object and GEO’s Knowledge Package but focuses on creating a reproducible basis out of the code and data so that the other research assets (i.e., virtual labs, web API services, and workflows) can be inferred from it. A special feature of the code component is its structuring into self-contained and reusable functions that fulfil a specific task (e.g. data transformation) instead of enabling access to the analysis script as a whole. The primary aim of the D2K-Package is to enhance the reusability of computational research outputs by enabling audiences with varying skill levels to follow the process from data analysis to knowledge generation.

In the following sections, we first outline the concept of the D2K-Package and show how researchers can develop a D2K-Package based on their reproducible analysis. Afterwards, we showcase its applicability using a hydrological use case, which we provide as a demonstrator so that the D2K-Package concept can be tested and explored. The paper concludes with a discussion on the integration of the D2K-Package into the research cycle, its benefits and limitations.

## Methods

### The concept of the Data-to-Knowledge Package

**Figure 1.**
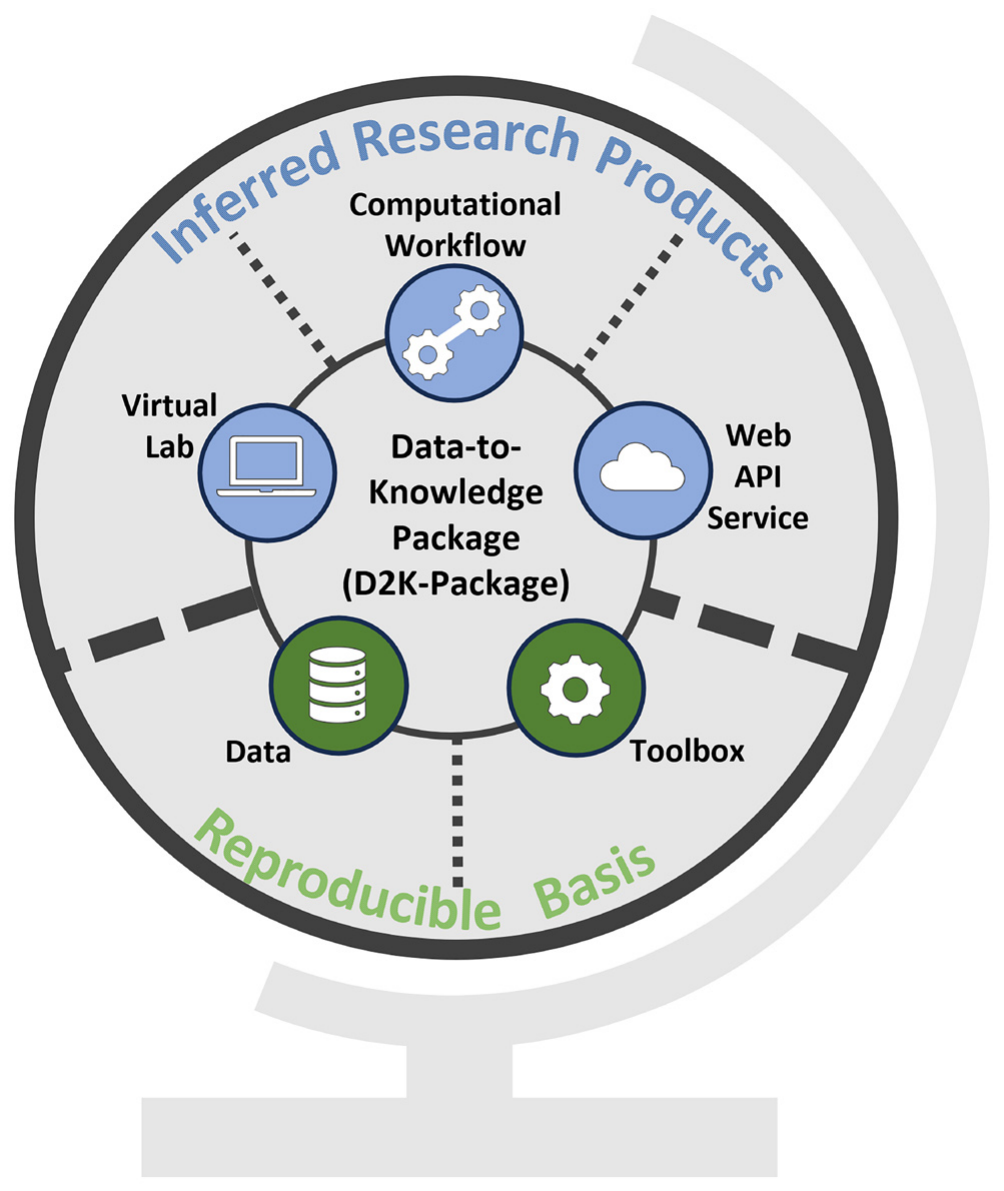
Conceptual overview of a Data-to-Knowledge-Package. Bottom: Reproducible basis composed of the *Data* & *Toolbox*. Top: Inferred research products built on top of the reproducible basis. All these components are linked in a Data-to-Knowledge Package.

A D2K-Package ( Figure 1) links to specific research assets to tackle key technical challenges encountered in a researcher's work:

- Verifying and reproducing the results presented e.g. in a publication needs access to the data and source code. The D2K-Package supports these tasks by linking to the ***Data*** and the ***Toolbox*** ( Figure 1), which together form the reproducible basis. In a Toolbox, the code is structured in such a way that the entire analysis is divided into self-contained and reusable functions that can be chained together to reproduce the analysis or called individually in a different context. More details on the creation of a Toolbox are provided below.
- Exploring the analysis and evaluating its relevance and reusability in other use cases requires restoring the computational environment. This involves tasks like installing a specific R ^32^ version with required packages or Docker to build and run images. However, this process is time-consuming and may be challenging or even unfeasible for users lacking the necessary technical skills, which may discourage them from attempting to run the analysis. The D2K-Package supports these efforts by linking to a ***Virtual Lab*** allowing users to engage more deeply with the code.
- Reusing parts of the analysis in a custom script requires copying the relevant code snippets and the necessary libraries, which can be an error-prone task. The D2K-Package overcomes this issue by linking to a ***Web API Service*** to which calls to the functions contained in the Toolbox can be sent from a source code script.
- Understanding the role of each function in an analysis, which input parameters are needed, and how the functions are connected requires familiarity with the programming language and effort. This is particularly challenging if the analysis script contains several thousand lines of code. To provide an entry point to the analysis and help other researchers (with and without coding skills) receive an overview of the analysis workflow composed of the functions and the configuration, the D2K-Package links to an executable and readily-sharable ***Computational Workflow***.

Consequently, a D2K-Package aims to facilitate various forms of reuse, including inspecting the analysis pipeline and running the analysis as-is. In addition, a D2K-Package places a particular emphasis on enabling reuse in new contexts by allowing users to customize the configuration of the analysis and extend it.

**Figure 2.**
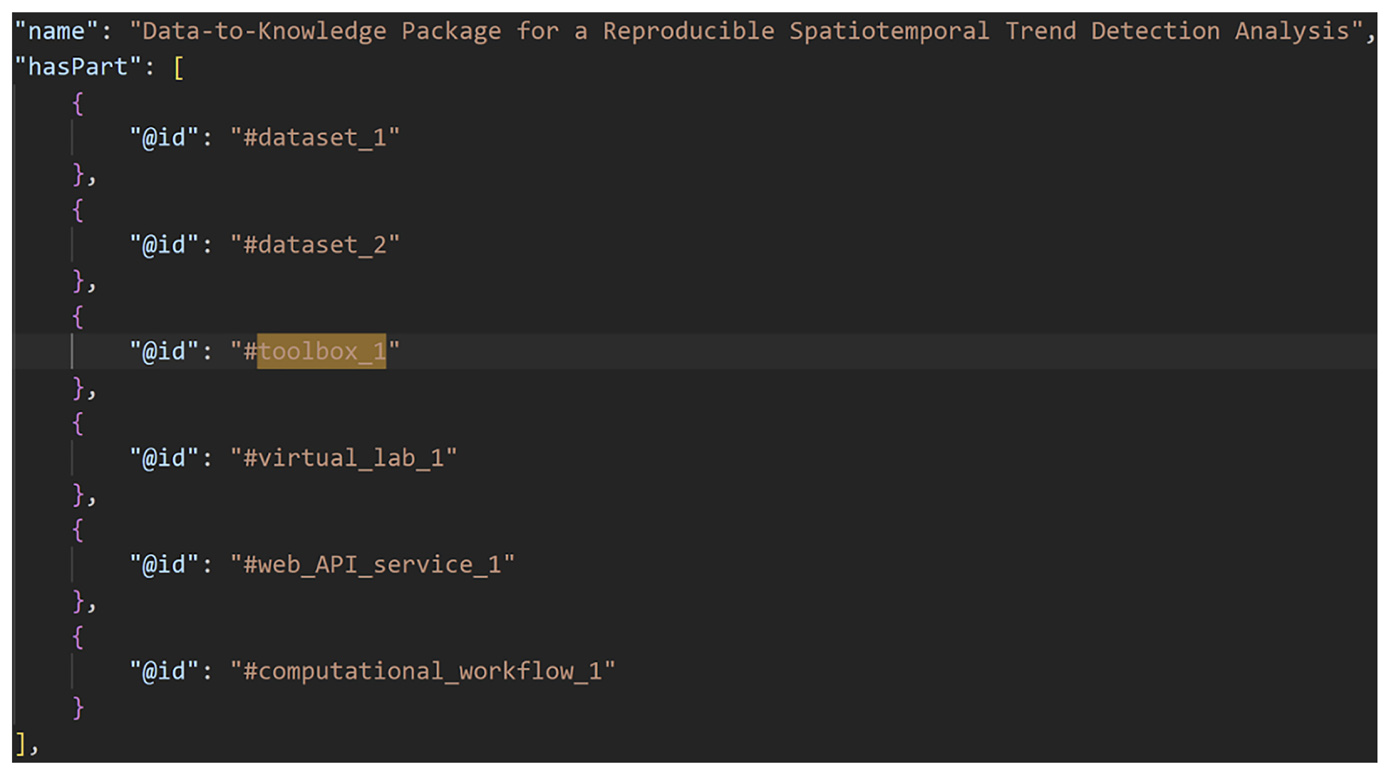
A Data-to-Knowledge Package’s ro-crate metadata file. Extract of a ro-crate metadata file (part 1) showing the components (“hasPart”) of a Data-to-Knowledge-Package.

The main feature of the D2K-Package is that it acts as an overarching meta-object including links to these components but it does not create another physical copy of them in a self-contained compendium as proposed in related approaches ^20, 33^. We chose this approach to avoid redundancy (e.g., copies of a given dataset) and possible synchronization issues caused by divergent code developments. Hence, several D2K-Packages can link to the same specific research asset (e.g., a dataset). Figure 2– Figure 4 show excerpts from a reference implementation of a D2K-Package in the form of a ro-crate metadata file ^25^. The file is a human- and machine-readable way of describing research assets of different types based on the structured data format JSON-LD and vocabularies, e.g. schema.org. The D2K-Package components *Data*, *Toolbox*, *Virtual Lab*, *Web API Service* and *Computational Workflow* are listed under the metadata element “ *hasPart*”. The “ *@id*” is a reference to the corresponding json object that contains the name, resource type and identifier (i.e. URL, DOI) of the D2K-Package component.

**Figure 3.**
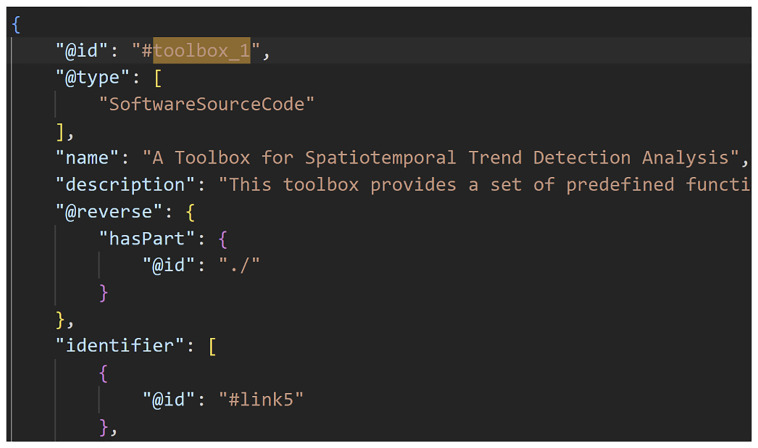
A Data-to-Knowledge Package’s ro-crate metadata file. Extract of a ro-crate metadata file (part 2) showing the Toolbox of a D2K-Package.

**Figure 4.**
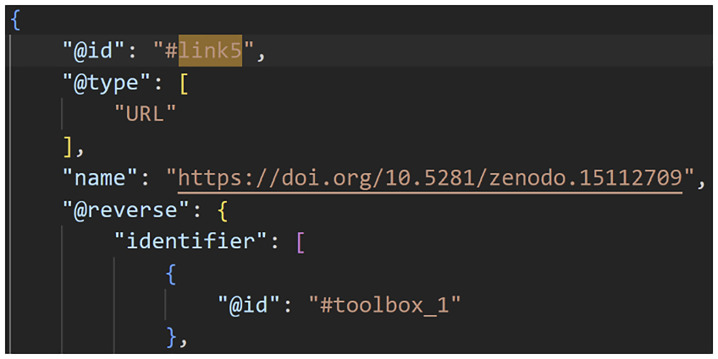
A Data-to-Knowledge Package’s ro-crate metadata file. Extract of a ro-crate metadata file (part 3) showing the link to the Toolbox component.

Ideally, the components are published as citable assets in repositories using permanent identifiers (e.g. a Digital Object Identifier, DOI) to which the D2K-Package can link ^34^. 

A D2K-Package can evolve and be expanded to include new materials in order to take account of the dynamic nature of publishing research assets. For example, a scientific paper under review can be supplemented by a D2K-Package and become part of the D2K-Package after publication. For this reason, the D2K-Package ro-crate file shown below should be published with, ideally versioned, DOIs. A D2K-Package can also refer to other research materials that facilitate the reusability of computational research. A poster, talk or similar can be a useful means of providing a detailed explanation of the context. Also, open educational resources (e.g. links to webinars, slides, tutorials) can contribute to a better understanding of the analysis and consequently facilitate reuse. Nevertheless, such creative outputs are not in the scope of this paper.

The following sections will elaborate on how the components can be created to achieve a D2K-Package that supports the reuse of computational research and the understanding of the path from data to knowledge generation. We refer to the *developer* who creates a D2K-Package and the *user* who works with it.

### Data

The data is generated by the developer of a D2K-Package or integrated from a third-party data provider. Technically, a D2K-Package can link to any data. Conceptually, the D2K-Package is supposed to encourage reuse. Hence, the data should fulfil at least some of the FAIR Principles and be released openly (data complying with the FAIR principles is not necessarily openly accessible ^35^). This means that the data should be described with rich metadata to ensure *findability*. In addition, *accessibility* should be granted by linking to each input data needed to reproduce the analysis. Ideally, the URL to the data is a permanent identifier (e.g., a DOI) to ensure the data is time-stamped, long-term accessible, and immutable. To ensure *interoperability*, the data should be stored in text-based formats (e.g., csv or json). Binary formats (e.g., GeoPackage, shapefile) are possible if they have an open specification.

Finally, the data should be released under an open license (ideally CC0 ^36^) to ensure *reusability*. Open licenses that restrict remixing, transforming, or building upon the data (e.g., CC-BY NoDerivatives ^37^) should not be used in a D2K-Package. These aspects become relevant at the latest when the D2K-Package is published. However, during the development of a D2K-Package, the data can be kept private.

### Toolbox

Besides the data, a D2K-Package links to a Toolbox created by the developer. The Toolbox contains the containerized analysis code (e.g., written in R or Python) used to generate the results in a research article and a specification of the computational environment. The source code is split into reusable functions that fulfil a specific task (e.g., processing data, running a statistical analysis). The functions follow the “input - processing - output” logic, meaning that each function takes some data (e.g., a table, figure) and a parameter configuration as inputs, runs the task on that data based on the configuration, and outputs the result (e.g., a table, figure). Furthermore, the functions are designed such that they do not depend on the other Toolbox functions and can be called individually. To ensure that the developer’s original computational environment can be recreated in the container, the Toolbox contains a specification of the runtime (e.g., R version) and a list of libraries and their versions.

A D2K-Package links to at least one Toolbox but it can reference other developers’ Toolboxes from which functions are reused. As with the data, the FAIR principles also apply to the Toolbox, considering software-specific characteristics ^18^ and the Toolbox should be made openly available at the latest when the D2K-Package is published.

The Toolbox and the data represent the central components in the concept of a D2K-Package and form the reproducible basis upon which the potential of reproducible research can be unlocked. In the following sections, we will see that virtual labs, web API services, and workflows can be created on top of the Toolbox without changing it.

### Virtual lab

In the context of this work, a virtual lab is a web application where the runtime and all software dependencies are installed in a ready-to-use programming instance, such as a JupyterLab environment providing a certain python runtime and all necessary packages in a predefined version. A D2K-Package links to such a virtual lab that is created by the developer of a D2K-Package based on the description of the computational environment contained in a Toolbox. Users can follow the link to the virtual lab and immediately start interacting with the code in the browser without the challenge of restoring the computational environment locally. They can develop the code further, try it out and change it in an executable environment, or use it in hands-on classes together with students. Since all users work in the same environment, differences in the results caused by differing library or runtime versions ^38, 39^ can also be avoided.

### Web API service

A web API service allows users to send requests to that service and receive a response via HTTP. Based on this request-response mechanism (also known as client-server communication), the developer of a D2K-Package can expose the functions in a Toolbox by setting up a server and deploying a web API service. The concept of a Toolbox can be seamlessly integrated into this mechanism since its functions are encapsulated, containerized, and well-defined regarding input and output. The web API service acts as a wrapper that enables immediate reuse of a Toolbox function. The wrapper takes the input information sent by the user (i.e., data, parameter configuration), executes the function, and outputs the result as part of the response for further processing in the user’s source code. Every function in the Toolbox is wrapped in such a way and has a dedicated endpoint that can be reached via a URL. A D2K-Package links to such a web API service provided by the developer of the D2K-Package.

### Computational workflow

A computational workflow is a chain of steps, each running a computational process. Such an analysis pipeline, which should be scripted according to the requirements in reproducible research, comprises steps such as data import, processing, analysis, and visualization. The concept of a Toolbox with its set of functions and the web API service exposing these functions seamlessly integrate with the idea of computational workflows. The developer of the D2K-Package can describe the analysis pipeline using a human-readable workflow language (e.g., Common Workflow Language). Such a workflow description can be created based on the Toolbox functions made available via the web API service, without having to change them. As the Toolbox functions are self-contained and independent, they can be extracted from the workflow context and combined with other functions. A workflow described in this form increases the reusability of the analysis by providing a quick overview and information on which functions are used in which order, the input parameters, and the outputs. The concrete implementation details of the functions are abstracted away from the user but can be explored in the virtual lab. A D2K-Package links to one or more computational workflows created by the developer of the D2K-Package. One can reuse the entire workflow or parts of it by combining it with functions used in other workflows.

## Results

We have applied the concept of the D2K-Package to a real research use case to demonstrate its applicability. In the following, we briefly present the hydrological use case and then describe the realization of the D2K-Package based on this use case.

### A hydrological use case

The use case explores hydrological dynamics by addressing changes in water optical properties in the Gulf of Riga — a semi-enclosed subbasin of the Baltic Sea. Changes in water transparency and colour in the Gulf of Riga are pressing ecological concerns ^40^, as they can significantly impact underwater habitats, biodiversity, and ecosystem services. However, the exact causes of these changes remain unclear, highlighting the need for a comprehensive investigation into the hydrological and biogeochemical processes driving these phenomena.

The use case is structured around several consecutive research questions aimed at exploring the interplay between land, freshwater, and marine ecosystems, with a focus on the River Daugava-Gulf of Riga continuum. For D2K-Package presentation purposes, we focus on the workflow addressing the first research question “ *Do the optical properties in the Gulf of Riga water change in the long term?”* by analysing long-term changes in water transparency in-situ measurements.

**Data:** The use case is based on two input datasets from third-party providers, which are both released under an open license but not under a permanent identifier. For reproducibility reasons, both input datasets are made available via Zenodo (see Data Availability) ^41^. One dataset is made available in geojson format and comes from an OGC API Features service ^42^. It can be directly downloaded from a URL and passed to a function in the Toolbox or to a web API service. In the context of the use case, this data holds information about long-term changes in water optical properties of the Gulf of Riga. The dataset includes key variables such as geographical coordinates (latitude and longitude), sampling station identifiers, sampling dates, and corresponding water transparency (Secchi depth, m) and water colour measurements (expressed in Forel-Ule scale). The D2K-Package contains a link to the OGC API Features service as the original data source and a DOI to the Zenodo record.

The second dataset is provided as a shapefile by the Baltic Marine Environment Protection Commission, which is also known as the Helsinki Commission (HELCOM) ^43^. This dataset includes information about assessment units for the Baltic Sea. The polygons are based on HELCOM open sea basins and are integrated with coastal water types as defined in the Water Framework Directive. The associated shapefile contains an attribute table with information, including country designation, subbasin identification (HELCOM_ID), subbasin level classification (e.g., Gulf of Riga), water type name (e.g., Gulf of Riga transitional waters), and additional data such as the area of the polygon.

For this specific dataset, users need to accept a data usage disclaimer before they can copy the download link and pass it to the function in the Toolbox. However, the download link becomes inactive after several minutes. Unfortunately, the inactive link does not redirect the user back to the page where they can accept the disclaimer again to reactivate the download link. Hence, it is disadvantageous to add the download link to the D2K-Package. Instead, we added the link to the website showing the data disclaimer, supplemented by the DOI to the Zenodo record.

**Toolbox:** The analysis pipeline developed in the use case is written in the open-source scripting language R ^32^. In the context of the use case, this analysis employs a workflow for data processing, trend analysis, and visualization. By identifying temporal and spatial patterns of environmental variables, the workflow supports the exploration of underlying links between hydrological processes and water optical properties in the Gulf of Riga.

We divided the analysis into seven functions, each accepting URLs to input datasets and producing outputs that, except for the last functions in the pipeline producing visualizations, are further used as inputs in the next function. Hence, also intermediate results can be investigated or used in different contexts. The functions and the specification of the computational environment are encapsulated in a Docker container and stored on GitHub. Docker is an open-source solution to build and run containerized applications ^44^ and has proven to be particularly useful in the context of reproducible research, for example, when it comes to handling complex dependencies ^29, 45^.

To specify the versions of the runtime and the libraries used in the Toolbox, we created an environment.yml file and used Anaconda as a package hosting service. To also take other reproducibility guidelines into account, the repository contains an open-source license to clarify usage (i.e., Apache License 2.0) and a README to document the content. A DOI-linked and citable version of the repository is made available on Zenodo, which was chosen because of its seamless integration with GitHub. The D2K-Package contains a DOI to the Zenodo record and a URL to GitHub.

**Virtual Lab:** We chose MyBinder to create a virtual lab based on the Toolbox. MyBinder is an open-source web application that recreates a computational environment based on a git repository containing information on the runtime, libraries and their versions, and the source code scripts ^46^. Users can engage with the code more deeply in a ready-to-use virtual lab providing the corresponding programming environment (in this case RStudio). The resulting virtual lab can be accessed and shared via a URL with other users and thus become part of a D2K-Package.

On the downside, MyBinder is a test instance with limited resources and does not guarantee to be operational when needed. However, it is also possible to use any other MyBinder web service or deploy and host an own instance.

**Web API service:** Interoperability is an essential pillar of the FAIR principles and the use of standards can help to achieve it. In order to further increase reusability and leverage community standards, the web API service was realized based on the OGC API Process standard ^47^. Such a process offers an endpoint to execute an algorithm via HTTP and is well-defined concerning input parameters and outputs. Usually, these processes take a link to the input data and also generate a link to the output data. Hence, the concept of an OGC API Process can be used to make the Toolbox functions available as a web API service. To implement the web API service based on the OGC API standard, we made use of pygeoapi, an open-source solution to deploy RESTful OGC API services ^48^. We have created an OGC API process file for each Toolbox function. The file is responsible for taking the input parameters and creating a Docker command to execute the function.

A D2K-Package contains a link to the OGC API service. From that link, users can receive a list of available processes (i.e., the Toolbox functions via GET /processes), execute a process (POST /{processId}/execution), and receive metadata (GET /processes/{processId}). The metadata also contains a link back to the GitHub repository containing the Toolbox and the corresponding Zenodo record enabling users to explore the underlying source code and cite the resource.

**Computational workflow:** An important selection criterion for the right workflow tool is the availability of a drag-and-drop workflow editor. Such a canvas can support users without programming knowledge in the development, execution and modification of readily-sharable workflows. For this requirement, the Galaxy platform ^31^ seems to be better suited than related software solutions such as Nextflow ^29^ and Snakemake ^30^. Galaxy is an actively maintained open-source web application that provides such a user-friendly interface. In addition, Galaxy has a large number of pre-installed tools, is extensible, has a large user community, and runs on a shared public cloud-based infrastructure.

**Figure 5.**
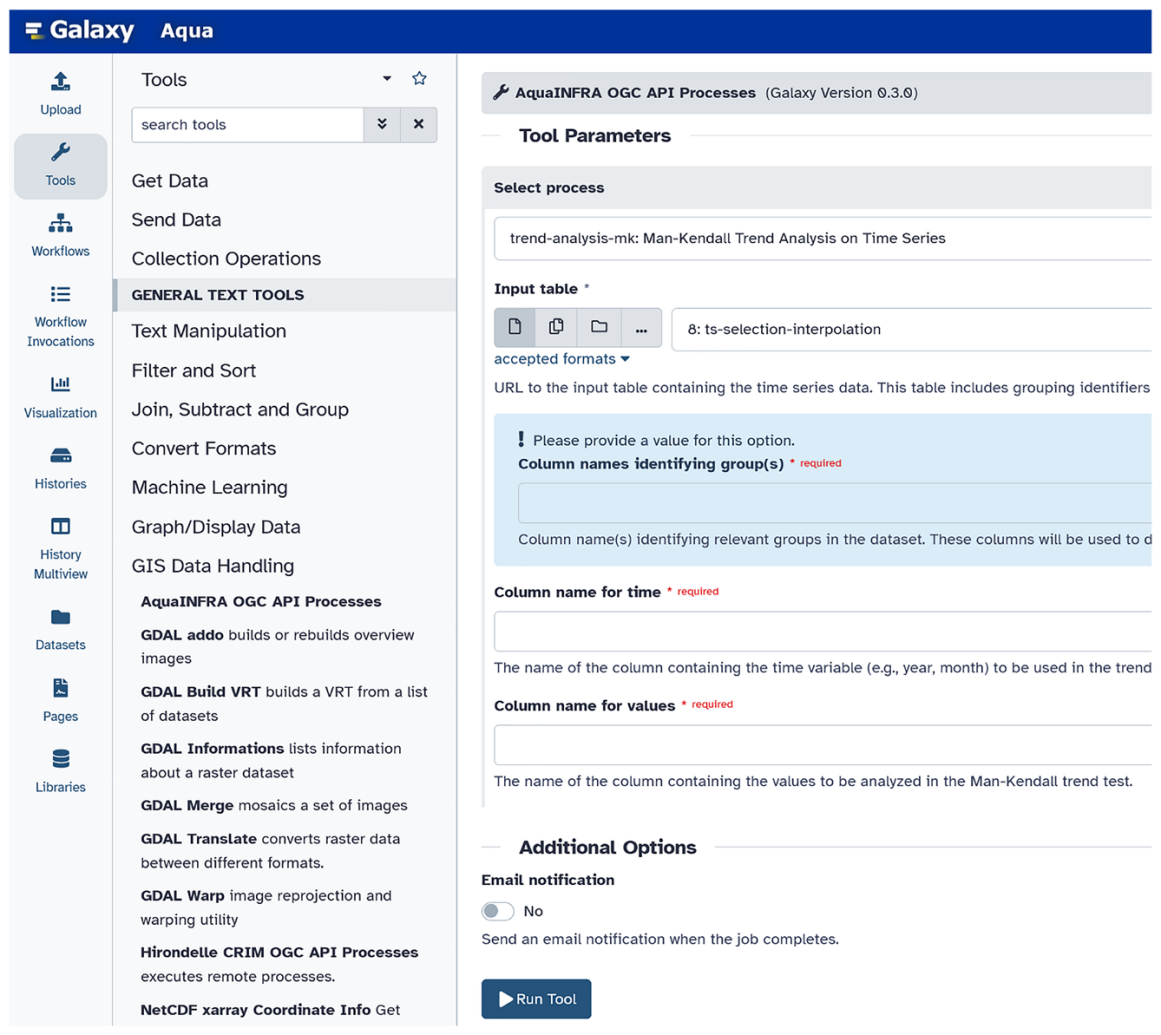
Integration of the OGC API Processes into the Galaxy platform ( link to Galaxy, link to the code). Users can find the tools we developed under GIS Data Handling – AquaINFRA OGC API Processes. Then, users can select the function from the dropdown list and complete the corresponding form, which is different for every function.

Integrating each Toolbox function separately as a tool into the Galaxy platform is possible but inefficient if the number of functions is large. For this reason, we developed a Galaxy tool that wraps the OGC API service and makes all exposed processes (i.e., each Toolbox function) available in one Galaxy tool (see Figure 5). Users can select the processes one after the other and fill the corresponding input parameters. The functions can then be connected to a workflow to show the entire analysis pipeline including all steps from data to knowledge generation (e.g., data input, pre/post-processing, analysis, and visualization).

**Figure 6.**
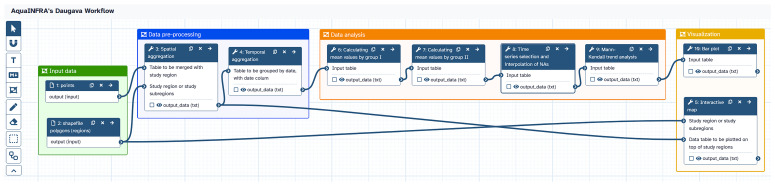
Galaxy workflow showing the analysis pipeline of the hydrological use case. The workflow is composed of seven steps (excluding input datasets, one function is used twice) and shows the entire analysis pipeline from data import to visualization.

Figure 6 shows the analysis pipeline of the hydrological use case implemented as a Galaxy workflow. The two datasets introduced above are used as inputs. The functions are executed one after the other, whereby the output of one function becomes the input of the subsequent function. Users can click on each function to check and modify the configuration and then re-run the workflow. The workflow can be shared with other users within the Galaxy platform and released on Zenodo. These two links are included in the final D2K-Package.

**ro-crate metadata file:** We created the ro-crate file for the D2K-Package using Describo ^49^, an open-source metadata editor for describing data. For the components of the D2K-Package, we reviewed the schema.org and bioschemas.org vocabularies to find the most appropriate resource types. As a result, we used “ Dataset” for the input data, “ SoftwareSourceCode” for the Toolbox, “ SoftwareApplication” for the MyBinder-based virtual lab, “ WebAPI” for OGC API-based web server, and “ ComputationalWorkflow” for the Galaxy-based workflow. We published the file on Zenodo, an open-source repository hosted by CERN providing versioned DOIs and a profound set of metadata. Research assets can be added to the D2K-Package creating a new version and a new DOI of the record. The DOIs of previous versions are still available.

### User interface

**Figure 7.**
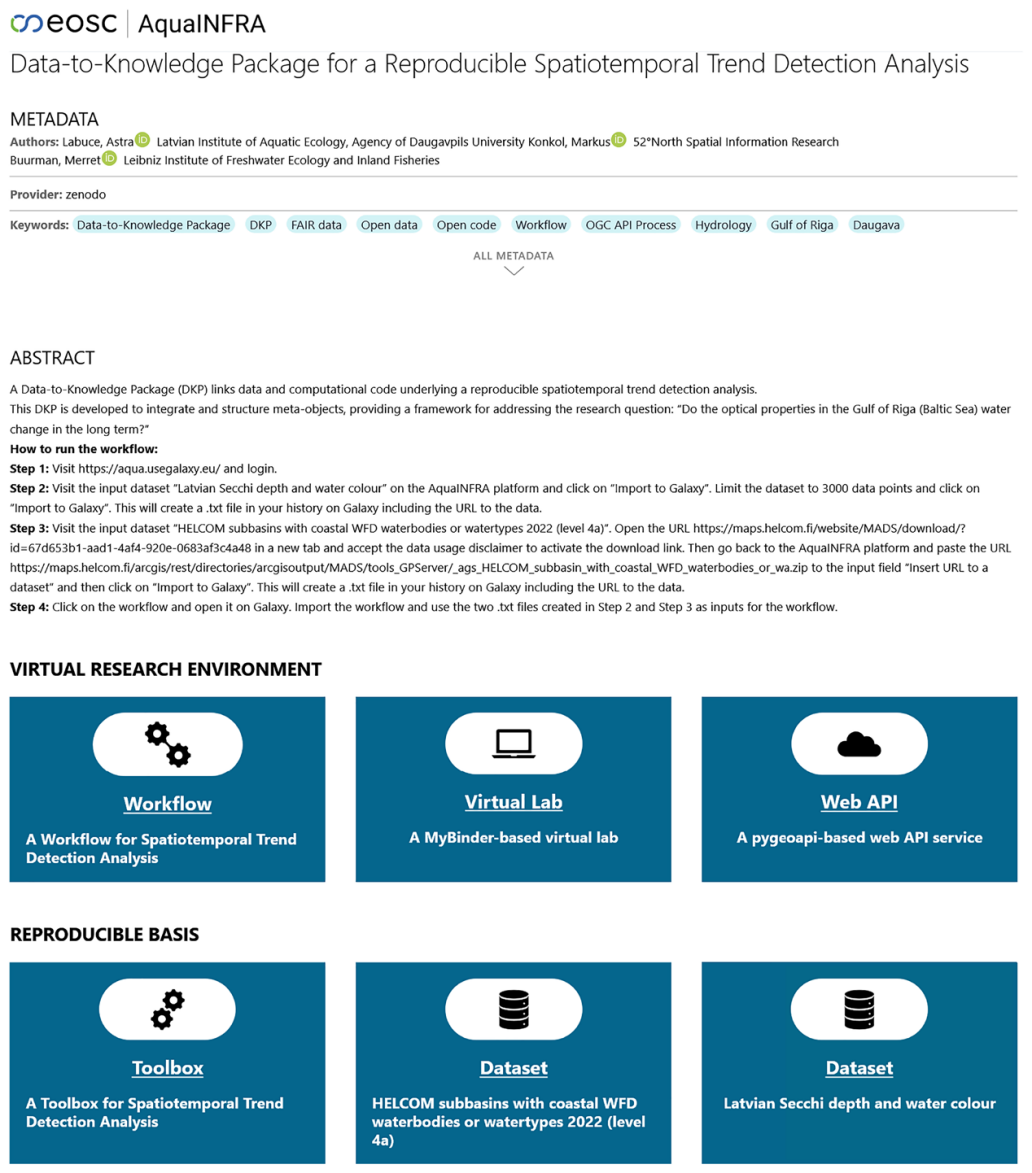
The landing page of a Data-to-Knowledge Package ( https://aquainfra.dev.52north.org/result/zenodo:15354757). Top: Metadata and a description. Middle: Virtual Research Environment linking to the workflow, virtual lab, and web API. Bottom: Reproducible basis linking to the toolbox and the input datasets.

Figure 7 shows a possible user interface for a D2K-Package. The landing page first shows the metadata such as title, authors, and a description. Below this is the so-called *Virtual Research Environment* (VRE), consisting of a Galaxy-based workflow, a MyBinder-based virtual lab and a pygeoapi-based web API service. The tile for the workflow was placed at the beginning, as it is intended to serve as the first entry point into the analysis. Clicking on it redirects the user to the Galaxy platform. Similarly, the virtual lab tile directs users to MyBinder and the Web API tile to the pygeoapi server. Below the VRE, users see the reproducible basis, consisting of input data tiles redirecting users to the website where they can find the download link and a Toolbox tile redirecting users to the GitHub repository.

## Discussion

The goal of the Data-to-Knowledge Package (D2K-Package) is to help researchers understand the path from data to knowledge generation and reuse open FAIR data and code. The D2K-Package aims to foster collaboration among researchers and facilitate knowledge dissemination, reducing redundant efforts and accelerating scientific progress. It incorporates reproducible code and data at its core, while providing multiple opportunities to understand and build upon the work. We applied the D2K-Package concept to a real research use case and demonstrated how the D2K-Package and its components (i.e., *Data*, *Toolbox*, *Virtual Lab*, *Web API Service* and *Computational Workflow*) can be designed. Next, the advantages of developing a D2K-Package are discussed, but the challenges and limitations are also critically examined.

### Research cycle

**Figure 8.**
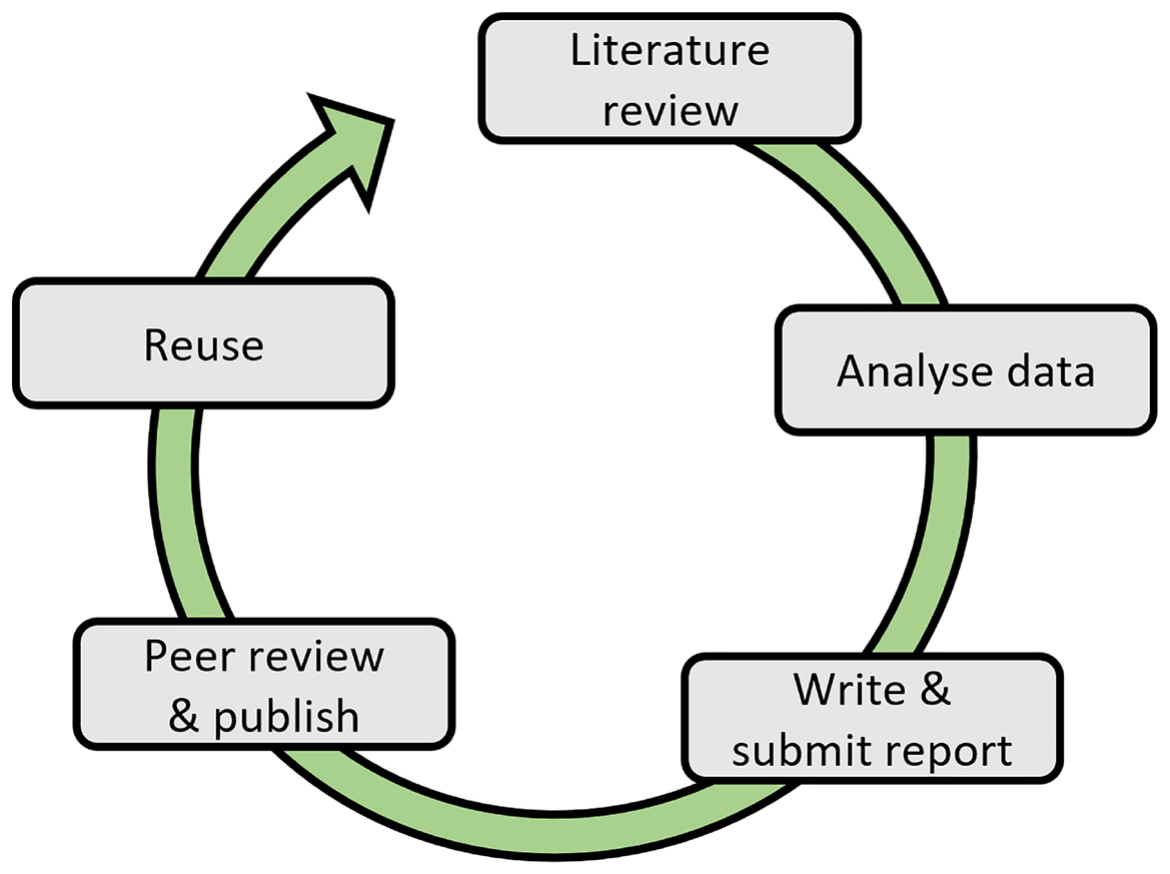
The research cycle. The research cycle shows the steps that can be supported by the Data-to-Knowledge Package.

The D2K-Package offers several opportunities to unlock the potential of a reproducible analysis throughout the research cycle (see Figure 8). The first entry point to the analysis is the workflow, which can help users understand the analysis and underlying configuration. By using tools such as Galaxy, users can re-run the workflow, modify it, or reuse parts of it in other contexts. This can be useful for researchers who are compiling a literature review at the start of a research project and trying to find related work to build on. Facilitating the reuse of an analysis entails the risk that the code will be reused without being scrutinized. The D2K-Package should not be understood as a shortcut to reusing research materials without having a thorough understanding of the implementation details. Instead, users can delve deeper into the code by exploring the virtual lab including the source code files. This virtual lab can be beneficial for other researchers who need a deeper understanding of the code before they can reuse parts of it. They do not have to spend time restoring the computational environment, but can start immediately. A researcher developing an analysis to answer a research question might want to reuse some of the functions provided by the Toolbox and can do so by integrating the remote OGC API Processes into the script. Experiencing the concrete benefits of this approach might incentivize researchers to develop a D2K-Package out of their own analysis before submitting the report to a conference or journal. Doing so can help them to ensure that the analysis is reproducible, verifiable, and reusable. Moreover, the resulting D2K-Package submitted for peer review can later be updated and expanded to also reference the peer-reviewed publication.

A D2K-Package, especially the workflow, can also be a helpful tool for reviewers who already have to invest a considerable amount of time in reviewing the paper and do not have the time to familiarize themselves with the analysis underlying the results of the paper. Users who do not have the necessary skills can also benefit from the workflow, as no knowledge of a programming language is required to follow the analysis, modify the underlying configuration, and use a different dataset (also known as “replication” ^50^).

### Use case

Applying the D2K-Package to the use case revealed a number of challenges. The input data can become an issue if users need to accept data usage disclaimers or login before they can access the download link. This is an issue with respect to machine-readability as it is not possible to use the download link in the code without manual action.

The analysis pipeline was almost completed by the researcher working on the use case when we started to apply the D2K-Package to it. As a result, it was necessary to change the code. For example, the entire code had to be split into individual reusable functions that follow the logic “input - processing - output” instead of having a series of lines of code that can be executed one after the other. Consideration also needed to be given to how the code could be split into functions and how small the functions should be. It is important to find the right balance to design functions that are small enough to be reused in other contexts while still performing a meaningful task. Ultimately, each developer decides for themselves when a function is reusable. In addition, developers need to think carefully about which input parameters should be passed to the function and what the intermediate results are. Such problems are well known in connection with the development of software libraries, but can still pose a major challenge for non-software developers. For a D2K-Package to be practicable, it should therefore be considered from the start in order to avoid too much work afterwards, which is anyway recommended to ensure reproducibility ^51^.

A persistent counterargument in connection with reproducible research is the time required for this. Based on our experience with the use case, it cannot be denied that this challenge also applies to the creation of a D2K-Package. Hence, it is unrealistic to expect that every kind of workflow is transformed into a D2K-Package, though a transformation is not required if the creation of a D2K-Package is considered from the beginning. Possible guiding questions to decide whether a D2K-Package is meaningful or not are:

- Should other researchers be able to reuse parts of the analysis in their own work?
- Should the analysis pipeline be presented as a workflow using, e.g., Galaxy?

If both answers are negative, the creation of a D2K-Package will probably not bring added value. However, if one or even both questions can be answered in the affirmative, a D2K-Package is a low-hanging fruit. The reason for this is that in order to create workflows or reusable code, the functions have to be developed in such a way that they can be called and executed individually anyway. In addition, containerization is necessary anyway to ensure executability. Consequently, the creation of a Toolbox is no longer a major additional expense.

Creating a MyBinder-based virtual lab is easy once a Toolbox has been created, as it contains all the necessary information. However, resolving dependency conflicts (e.g. incompatible library versions) can be a challenge. Moreover, a Toolbox also makes it easier to provide OGC API processes, as these only need to execute the containerized functions. Integrating the functions into the Galaxy platform, on the other hand, requires a certain amount of additional work, as the necessary tools have to be created.

### Persistence

Making the D2K-Package persistent can be achieved by publishing it in a repository that assigns DOIs to its records. It is more difficult to ensure the persistence of the individual components linked from a D2K-Package. Ideally, all components generated by the developer of the D2K-Package are published in a repository offering DOIs. A Toolbox maintained on GitHub can be published permanently via the GitHub-Zenodo integration. The Galaxy workflow and the source code files responsible for the OGC API Processes can also be published on Zenodo.

However, we have seen in the hydrological use case that the data may only be available under a URL and not a DOI. Therefore, if the data is not maintained in the long term, either the data or the link to the data may change over time, which affects reproducibility. One way to mitigate this problem is to download the data and publish it in a repository such as Zenodo, if the license allows it. This approach would facilitate access and assign a permanent identifier to the data. However, this approach leads to duplication and is only possible if the file size restriction of the repository allows it, which is usually not the case for big data. This limitation is not specific to the D2K-Package, but shows where the idea of reproducible research and FAIR and open data conflicts with reality. Furthermore, the problem is not an exceptional case, but can always occur if the researcher has not generated the data and has no control over it. The responsibility lies with the data providers, who must provide open and FAIR data that is also machine-readable.

### Portability

The D2K-Package is designed to facilitate the portability of individual components to ensure sustainability and longevity. The code is encapsulated in a Docker container, which can be deployed wherever needed and maintained on any web application based on git, such as GitHub or GitLab. The MyBinder instance used to realize the Virtual Lab is a test instance. It is not guaranteed to work at all times and has limited resources, which reduces its capacity when many users access the instance simultaneously. Nevertheless, the Toolbox is not tied to a specific MyBinder deployment but can run on any other instance offering the same functionality.

A similar problem exists with the pygeoapi-based web API service and the Galaxy platform which require a server. While the Galaxy tool wrapping the OGC API Processes can only run on a Galaxy platform, it is not bound to a particular Galaxy instance. Every operator hosting a Galaxy instance can also run that tool. Thus, also the workflows can be run on different Galaxy instances. We see the responsibility for providing server capabilities not with the researchers but with those who have reproducibility as a requirement, such as funders and publishers. Initiatives like the European Open Science Cloud and their concept of a “Node” can be a promising opportunity to provide access to such tools and services. Yet, the realization of the D2K-Package in this work is just a reference implementation. It can be realized using different technologies. The workflows, for instance could also be realized using other tools depending on the user requirements, such as Nextflow ^29^ to address experienced programmers.

In addition to sustainability, the development of a portable solution is advantageous in the case of big data. For example, the Toolbox could be deployed where the data is stored to avoid data transfer.

## Ethics and consent

Ethical approval and consent were not required.

## Data Availability

Zenodo: Encouraging reusability of computational research through Data-to-Knowledge Packages - A hydrological use case (Input data) ^41^. https://doi.org/10.5281/zenodo.15234377 The analysis used in the hydrological use case takes two input datasets coming from a third-party data provider released under an open license. The first input dataset ^43^ can be downloaded after accepting the data usage disclaimer. The second input dataset ^42^ of the workflow can be downloaded from an OGC API Features service. All datasets are released under an open license (Creative Commons Attribution 4.0 International).

## Software availability

Zenodo: Data-to-Knowledge Package ^52^. https://doi.org/10.5281/zenodo.15354757

Zenodo: A Toolbox for Spatiotemporal Trend Detection Analysis ^53^. https://doi.org/10.5281/zenodo.15350797

Zenodo: Galaxy workflow ^54^. https://doi.org/10.5281/zenodo.15350861

Zenodo: Source code for the user interface ^55^. https://doi.org/10.5281/zenodo.15129277

Zenodo: Galaxy tool ^56^. https://doi.org/10.5281/zenodo.15234678

GitHub: A Toolbox for Spatiotemporal Trend Detection Analysis: https://github.com/AstraLabuce/aquainfra-usecase-Daugava/tree/containerize

Galaxy workflow: https://aqua.usegalaxy.eu/u/markus.konkol/w/copy-of-adj-aquainfras-daugava-workflow-shared-by-user-alabuce

Galaxy tool: https://github.com/galaxyecology/tools-ecology/tree/master/tools/aquainfra_ogc_api_processes

User interface: https://aquainfra.dev.52north.org/result/zenodo:15354757

OGC API server: https://aquainfra.ogc.igb-berlin.de/pygeoapi/

All software outputs are released under an open license (Apache License 2.0).

## References

[1] Tollefson J Kozlov M Witze A : Trump’s siege of science: how the first 30 days unfolded and what’s next. *Nature.* 2025;638(8052):865–867. 10.1038/d41586-025-00525-1 39979570

[2] Matthews D : Far-right governments seek to cut billions of euros from research in Europe. *Nature.* 2024;635(8037):15–16. 10.1038/d41586-024-03506-y 39468345

[3] Costello MJ : Motivating online publication of data. *BioScience.* 2009;59(5):418–427. 10.1525/bio.2009.59.5.9

[4] Goodman SN Fanelli D Ioannidis JPA : What does research reproducibility mean? *Sci Transl Med.* 2016;8(341): 341ps12. 10.1126/scitranslmed.aaf5027 27252173

[5] Stodden V Bailey DH Borwein J : Setting the default to reproducible: reproducibility in computational and experimental mathematics.2013. Reference Source

[6] Konkol M Kray C Pfeiffer M : Computational reproducibility in geoscientific papers: insights from a series of studies with geoscientists and a reproduction study. *Int J Geogr Inf Sci.* 2019;33(2):408–429. 10.1080/13658816.2018.1508687

[7] Culina A van den Berg I Evans S : Low availability of code in ecology: a call for urgent action. *PLoS Biol.* 2020;18(7): e3000763. 10.1371/journal.pbio.3000763 32722681 PMC7386629

[8] Hutson M : Artificial Intelligence faces reproducibility crisis. *Science.* 2018;359(6377):725–726. 10.1126/science.359.6377.725 29449469

[9] Baker M : 1,500 scientists lift the lid on reproducibility. *Nature.* 2016;533(7604):452–454. 10.1038/533452a 27225100

[10] McCullough BD McGeary KA Harrison TD : Do economics journal archives promote replicable research? *Can J Econ.* 2008;41(4):1406–1420. 10.1111/j.1540-5982.2008.00509.x

[11] Collberg C Proebsting TA : Repeatability in computer systems research. *Commun ACM.* 2016;59(3):62–69. 10.1145/2812803

[12] Gil Y David CH Demir I : Toward the geoscience paper of the future: best practices for documenting and sharing research from data to software to provenance. *Earth Space Sci.* 2016;3(10):388–415. 10.1002/2015EA000136

[13] Bahaidarah L Hung E de Melo Oliveira AF : Toward reusable science with readable code and reproducibility. *International Conference on e-Science.* 2022;437–439. 10.1109/eScience55777.2022.00079

[14] European Commission: Open science in horizon Europe. Reference Source

[15] Stark PB : Before reproducibility must come preproducibility. *Nature.* 2018;557(7707):613. 10.1038/d41586-018-05256-0 29795524

[16] Trust but verify. *Nat Mater.* 2024;23(1):1. 10.1038/s41563-023-01790-z 38172553

[17] Wilkinson M Dumontier M Aalbersberg JJ : The FAIR guiding principles for scientific data management and stewardship. *Sci Data.* 2016;3: 160018. 10.1038/sdata.2016.18 26978244 PMC4792175

[18] Barker M Hong NPC Katz DS : Introducing the FAIR Principles for research software. *Sci Data.* 2022;9(1): 622. 10.1038/s41597-022-01710-x 36241754 PMC9562067

[19] Sandve GK Nekrutenko A Taylor J : Ten simple rules for reproducible computational research. *PLoS Comput Biol.* 2013;9(10): e1003285. 10.1371/journal.pcbi.1003285 24204232 PMC3812051

[20] Gentleman R Lang DT : Statistical analyses and reproducible research. *J Comput Graph Stat.* 2007;16(1):1–23. 10.1198/106186007X178663

[21] Chirigati F Rampin R Shasha D : ReproZip: computational reproducibility with ease. *Proceedings of the 2016 International Conference on Management of Data (SIGMOD '16)*. Association for Computing Machinery,2016. 10.1145/2882903.2899401

[22] Seamless sharing and peer review of code. *Nat Comput Sci.* 2022;2(12): 773. 10.1038/s43588-022-00388-w 38177390

[23] Alves AN Oliveira MM Koyama T : Ecdysone coordinates plastic growth with robust pattern in the developing wing. *eLife.* 2022;11: e72666. 10.7554/eLife.72666 35261337 PMC8947767

[24] Bechhofer S Buchan I De Roure D : Why linked data is not enough for scientists. *Future Gener Comput Syst.* 2013;29(2):599–611. 10.1016/j.future.2011.08.004

[25] Soiland-Reyes S Sefton P Goble C : Packaging research artefacts with RO-Crate. *Data Sci.* 2022;5(2):97–138. 10.3233/DS-210053

[26] Group on Earth Observation: Knowledge package. Reference Source

[27] Shakil A Lutteroth C Weber G : CodeGazer: making code navigation easy and natural with Gaze input. *Proceedings of the 2019 CHI Conference on Human Factors in Computing Systems (CHI '19)*.2019;1–12. 10.1145/3290605.3300306

[28] Nordmann E McAleer P Toivo W : Data visualization using R for researchers who do not use R. *Adv Methods Pract Psychol Sci.* 2022;5(2). 10.1177/25152459221074654

[29] Di Tommaso P Chatzou M Floden EW : Nextflow enables reproducible computational workflows. *Nat Biotechnol.* 2017;35(4):316–319. 10.1038/nbt.3820 28398311

[30] Mölder F Jablonski KP Letcher B : Sustainable data analysis with Snakemake [version 2; peer review: 2 approved]. *F1000Res.* 2021;10:33. 10.12688/f1000research.29032.2 34035898 PMC8114187

[31] Galaxy Community: The Galaxy platform for accessible, reproducible, and collaborative data analyses: 2024 update. *Nucleic Acids Res.* 2024;52(W1):W83–W94. 10.1093/nar/gkae410 38769056 PMC11223835

[32] R Core Team: R: a language and environment for statistical computing. R Foundation for Statistical Computing,2025. Reference Source

[33] Nüst D Konkol M Schutzeichel M : Opening the publication process with executable research compendia. *D-Lib Magazine.* 2017;23(1/2). 10.1045/january2017-nuest

[34] Nosek BA Alter G Banks GC : SCIENTIFIC STANDARDS. Promoting an open research culture. *Science.* 2015;348(6242):1422–1425. 10.1126/science.aab2374 26113702 PMC4550299

[35] Higman R Bangert D Jones S : Three camps, one destination: the intersections of research data management, FAIR and open. *Insights: the UKSG Journal.* 2019;32(1):18. 10.1629/uksg.468

[36] Hrynaszkiewicz I Cockerill MJ : Open by default: a proposed copyright license and waiver agreement for open access research and data in peer-reviewed journals. *BMC Res Notes.* 2012;5: 494. 10.1186/1756-0500-5-494 22958225 PMC3465200

[37] Creative Commons: Attribution-NoDerivatives 4.0 International. Reference Source

[38] Piccolo SR Frampton MB : Tools and techniques for computational reproducibility. *GigaScience.* 2016;5(1): 30. 10.1186/s13742-016-0135-4 27401684 PMC4940747

[39] Garijo D Kinnings S Xie L : Quantifying reproducibility in computational biology: the case of the tuberculosis drugome. *PLoS One.* 2013;8(11): e80278. 10.1371/journal.pone.0080278 24312207 PMC3842296

[40] Aigars J Suhareva N Cepite-Frisfelde D : From green to brown: two decades of darkening coastal water in the Gulf of Riga, the Baltic Sea. *Front Mar Sci.* 2024;11: 1369537. 10.3389/fmars.2024.1369537

[41] Konkol M : Encouraging reusability of computational research through Data-to-Knowledge Packages - A hydrological use case (Input data). *Zenodo.* 2025. 10.5281/zenodo.15234377 PMC12334915 40786725

[42] Latvian Secchi depth and water colour. AquaINFRA data discovery and access service. 2025. https://vm4072.kaj.pouta.csc.fi/ddas/oapif/collections/lva_secchi/items?f=json&limit=5871

[43] HELCOM subbasins with coastal WFD waterbodies or watertypes 2022 (level 4a). The Baltic Marine Environment Protection Commission (HELCOM), Helsinki, Finland, 2022. https://maps.helcom.fi/website/MADS/download/?id=67d653b1-aad1-4af4-920e-0683af3c4a48

[44] Merkel D : Docker: lightweight Linux containers for consistent development and deployment. *Linux J.* 2014;239(2): 2. Reference Source

[45] Boettiger C : An introduction to Docker for reproducible research. *SIGOPS Oper Syst Rev.* 2015;49(1):71–79. 10.1145/2723872.2723882

[46] Bussonnier M Forde J Freeman J : Binder 2.0 - Reproducible, interactive, sharable environments for science at scale. *Proceedings of the 17th Python in Science Conference.* 2018. 10.25080/Majora-4af1f417-011

[47] Open Geospatial Consortium: OGC API - Processes. Reference Source

[48] Kralidis T Webb B Tzotsos A : geopython/pygeoapi: 0.19.0 (0.19.0). *Zenodo.* 2025. 10.5281/zenodo.14592499

[49] La Rosa M : Describo. 2025. Reference Source

[50] The Turing Way Community: The turing way: a handbook for reproducible, ethical and collaborative research (1.0.2). *Zenodo.* 2022. 10.5281/zenodo.7625728

[51] Alston JM Rick JA : A beginner's guide to conducting reproducible research. *Bull Ecol Soc Am.* 2021;102(2): e01801. 10.1002/bes2.1801

[52] Labuce A Konkol M Buurman M : Data-to-Knowledge package for a reproducible spatiotemporal trend detection analysis (1.4). *Zenodo.* 2025. 10.5281/zenodo.15354757

[53] Labuce A Konkol M Buurman M : A toolbox for spatiotemporal trend detection analysis (1.0.1). *Zenodo.* 2025. 10.5281/zenodo.15350797

[54] Labuce A Konkol M Buurman M : A workflow for spatiotemporal trend detection analysis (1.0.1). *Zenodo.* 2025. 10.5281/zenodo.15350861

[55] Konkol M : AquaINFRA interaction platform v1.0.1 (1.0.1). *Zenodo.* 2025. 10.5281/zenodo.15129277

[56] Konkol M : AquaINFRA OGC API processes to galaxy (1.0). *Zenodo.* 2025. 10.5281/zenodo.15234678

