# Supplementary material for: Encouraging reusability of computational research through Data-to-Knowledge Packages - A hydrological use case
Source: Open Res Eur. 2025 Sep 22;5:123. Originally published 2025 May 6. [Version 3] doi: 10.12688/openreseurope.20221.3 (PMC12334915; doi:10.12688/openreseurope.20221.3)
Supplement: Peer review history

# Reviewer report: Reviewer response for version 2

**DOI:** 10.21956/openreseurope.22545.r56724

**Published:** 2025-09-02

**Authors:** David Tarboton, David Rosenberg

**Affiliations:** 1 Civil and Environmental Engineering, Utah State University, Logan, Utah, USA

**Failed Attempt to Reproduce Results in “Encouraging reusability of computational research through Data-to-Knowledge Packages - A hydrological use case.”** **https://doi.org/10.12688/openreseurope.20221.2** **.**

**Reviewer Report**

 David Rosenberg, David Tarboton

**General comments**

 This manuscript presents “data to knowledge packages” as a solution to encourage reusability and reproducibility of computational research in hydrology.  The manuscript is well written. The work is needed. The descriptions of elements of reproducibility are compelling. The key component is a web platform (GALAXY) that allows users to undertake common data management, import, analysis, and visualization activities. These activities include:

- Spatial aggregation: Assigns in situ environmental data points to spatial units based on polygon boundaries
- Temporal aggregation: Groups data points by time intervals for structured trend analysis
- Time Series Processing and Selection such as calculating mean values by group, selecting time series with sufficient data and interpolating missing values (NAs) to generate structured time series datasets optimized for trend detection.
- Trend Detection: such as Mann-Kendall analysis for statistically assessing long-term trends in environmental parameters across spatial units.
- Visualization: Interactive map generation for visualizing in situ data points over spatial assessment units, Trend representation in a static bar plot summarizing detected significant trends.

The manuscript aims to present a hydrological use case for the Gulf of Riga. The motivating question is: *Do the optical properties in the Gulf of Riga water change in the long term*?

**We find that the manuscript falls short in the following aspects:**

1. The hydrological use case is not actually given.
2. The web platform (GALAXY) and tools presented are too complex and fragile and end up being a barrier to, rather than helpful towards reproducibility and reusability.
3. We were unable to get the tools to work.
4. We could not see the analysis or results.
5. We did not gain any (new) understanding of the data , hydrologic system, or management of the system.

Thus our overall finding is that while the manuscript encourages reusability and reproducibility, the work was not reproducible itself. We believe it will take a major redesign of how the data, web platform, tools, analysis, and workflow engine are integrated to achieve the reusability and reproducibility goals. The paper fails to offer a contribution either in technical substance, reusability, or reproducibility. There needs to be significant attention to leading and helping a reuser/reproducer establish or gain access to the run time environment and from there on for the process to be automated, while at the same time transparent for this to become a helpful contribution.

**A framework for assessing research reusability and reproducibility**.

 We find this framework helpful for assessing research reproducibility and reusability:

1. Publicly available. Research artifacts should be in a public repository with persistent identifiers (preferably DOI’s).
2. Documentation. Writing should overview the project, explain the contents, and list results the authors intend to be reproduced. The documentation should be in human readable file format that will persist (e.g., text or markdown).
3. Organization. Distinguish between Data, Digital Artifacts that operate on Data, and Author generated results.
4. Computational requirements. Provide requirements and instructions to create or set up the run-time environment for the analysis.
5. Workflow. Provide instructions to use artifacts in the runtime environment to generate results.
6. Independent check. A non-author follows the prior steps to generate and validate the authors results.

The first 5 steps address findability, accessibility, interoperability, and reusability (FAIR) principles, while step 6 validates reproducibility.

 Steps (1) through (3) are generally well done in this paper.  The digital artifacts are available with a persistent digital object identifier in Zenodo (step 1) and included human-readable markdown (README.md) or Text (.txt) files that overviewed the purpose and explain the organization of the contents (Step 2). Across the project, the digital artifacts are nicely separated into data and the software that operates on the data (Step 3).

 Our concerns with this paper rest with steps (4) to (6). The complex technical requirements for reproducing what should be standard statistical analysis in an open-source procedural programming language is daunting and seems unnecessary. 

**Suggestions to improve**

1. The paper should show the key computational outputs and results in maps, figures, tables, etc., so that someone intending to reproduce or reuse this work knows what they are looking for, and so that the motivating question is answered.
2. Simplify the technological requirements. It seems that the work of this paper could be done solely in an open-source procedural language such as Python or R.
3. Make a well documented script that automates all steps of the workflow, including presentation of results.
4. Use a computational environment that will persist. For example, web platforms such as GALAXY may go away, change, or be inaccessible to the reproducer.
5. Provide guidance on more general approaches for the work to be reproduced.
6. Provide instructions to recreate the run time environment, specifically for Docker and Binder. Explain how a person trying to reproduce the work should set up these tools on their computer, particularly on one or more common platforms (Windows/Mac/Linux).
7. Aggregate all the components needed to undertake this work in a single public repository with digital object identifier so that users can find everything in one place. Perhaps the data to knowledge package ( https://doi.org/10.5281/zenodo.15354757) is this repository, but make this clear and lead a user on when they get to this.

Below we provide more detailed notes on the technical efforts by each of us to work with the web platform, data, and code provided. These notes can help identify where reproducibility broke down.

**Writing**

 Shrinking research budgets are not in our opinion the leading motivator for reproducible research. The leading motivator is trust in the findings brought about by seeing and understanding the rationale for the findings.  We suggest not opening the paper with a statement about shrinking research budgets.

 The paper states that a major obstacle is the “perception” that publishing reproducible results is time consuming.  This is not a just perception, it is real. 

**Notes on technical reproducibility efforts by Rosenberg**

Source Data

**1. Zenodo: Encouraging reusability of computational research through Data-to-Knowledge Packages - A hydrological use case (Input data)** **^41^** **.** **https://doi.org/10.5281/zenodo.15234377**

1. I was able to access and download both data sets.
2. This is simply a json file with no meta data or explanation of contents. Insufficient meta data.
  1. No instructions, interpretation on the Zenodo repository.
  2. No explanation of Latvian Secchi depth, units of measurement. Human readable format
3. Similar in the HELCOM dataset.

Software availability

**a. Zenodo: Data-to-Knowledge Package** **^57^** **.** **https://doi.org/10.5281/zenodo.15354757**

1. Good directions and meta data.
2. No explanation of the file ro-crate-metadata.json.

**b. Zenodo: A Toolbox for Spatiotemporal Trend Detection Analysis** **^58^** **.** **https://doi.org/10.5281/zenodo.15350797**

1. Repository with DOI – good. Also excellent description of contents in the meta data for the repository.
2. README.md file provides a good overview and explanation of the contents.
3. I followed the link on line 4 to the Binder. This is my first time using Binder. The link opened the following window:
4. What do I do next???
5. Poking around, I found my way to a virtual RStudio Server webpage that looks a lot like the R Studio platform I use a lot on my desktop. Cool!!
6. I was unclear which file to open first.
7. So I opened the map_shapefile_points.R.
8. I selected all the code and ran it. That generated the following figure and error (see red arrow at bottom)
9. At this point I gave up.
10. Back in the README.md file that I downloaded from Zenodo onto my desktop, starting on line 6, I found the ordering of the sections in the README.md file confusing. It seems the last section, “Create conda environment” needs to happen first?? Order the remaining sections in the order of the steps to reproduce the results.
11. So I tried the following: I opened a cmd prompt (I have windows 11) and was able to execute the 3 lines of code at the bottom of the README.md file under the section “Create conda environment.”
12. The last command executed but gave a bunch of warnings such as “could not solve for environment specs” and “The following packages are incompatible”  Can one still continue even with these errors/warnings?
13. Next I tried to the build the docker image. I continued in the same virtual environment and entered the first line of code in under the section “Building Docker image.” I got an error about unable to run the git command even though I have Git Desktop installed on my machine that I regularly use.
14. I got the same error when I tried to run in the root cmd prompt outside the environment.
15. I stopped here. There is evidently specialized knowledge or directions needed to execute these commands. Please add more directions/instructions.
16. At this point I was also unclear about the order or other steps needed to run the R code in src/ directory, the .json and .py files in the directory src/ogc_api_processes
17. For example, I ran the file test_post_requests.py in the folder pygeoapi_documentation which executed. But the window closed quickly before I could see any output. When I opened the same file in a text editor I could see generally a bunch of code, but it was hard to understand what is actually happening (or supposed to happen). Suggestions to improve:
  1. In the header provide an overview of the steps taken in the code.
  2. For each code section provide more information as comments about what is happening.
18. I opened the example_results folder and found a bunch of csv files. Excellent as this is a common and persistent data sharing format. I opened two of the output files. These can be improved by providing more meta data about the contents of the columns and units of measurement. It will also help to show the mapping between code files in the other folders and the results files. Which code file/section generates which result file?
19. OGC processes – I did not follow this section. It would help to organize this section as the steps the user needs to take.

**c. Zenodo: Galaxy workflow** **^59^** **.** **https://doi.org/10.5281/zenodo.15350861**

1. I was able to download and extract the files in this repository.
2. The README.md file had nothing in it.
3. Some of the txt files were either empty, had a short instance such as “{"galaxy_export_version": "2"} “, or had a long line of indecipherable code.
4. I stopped here.

**d. Zenodo: Source code for the user interface** **^60^** **.** **https://doi.org/10.5281/zenodo.15129277**

1. It looks like all the code is here, but there is not meta data in the Zenodo resource or explanation of the contents.
2. Nonetheless, I downloaded and opened the zip file.
  1. The README.md in the root folder provides a nice summary of the platform. Under Installation, I ran into the same error when I tried to execute the first command git clone https:// as above.
  2. The README.md in the docs folder looks like a nice organization of the user guide.
  3. I appreciated the instructions in the GettingStarted.md but did not follow them because they were long and required a lot of manual entries.

**Notes on technical reproducibility efforts by Tarboton**

 This is a record of the work I did to attempt to reproduce the results.

 I started from the D2K package given at https://doi.org/10.5281/zenodo.15354757. I tried to follow the steps given (given steps in italics, my comments in plain text):

***Step 1:*** *Visit* *https://aqua.usegalaxy.eu/* *and login.*

 I was able to create an account. The dependency on a specific site like this is a limitation. This site may be updated and the procedure may no longer work.

***Step 2:*** *Visit the input dataset “Latvian Secchi depth and water colour” on the AquaINFRA platform and click on “Import to Galaxy”. Limit the dataset to 5871 data points and click on “Import to Galaxy”. This will create a .txt file in your history on Galaxy including the URL to the data.*

 How to achieve this is not clear from the statement. I went back to the paper and guessed based on aquainfra being in the URL that the platform was what was given in the paper as *User interface:* *https://aquainfra.dev.52north.org/result/zenodo:15354757* This has the same set of instructions, but a search bar at the top.  I guessed and put in.  One dataset was located



 I clicked on this and clicked on Import to Galaxy.  I set the limit data points to 5871 and clicked on the next Import to Galaxy button.  Nothing happened.  I then clicked Copy URL



 Then in Galaxy I clicked “Upload”



 (noting the confusion related to terminology that the Upload button says Download from URL)

 And after stumbling around I found a place to Paste/Fetch data where I could enter the URL



 After clicking Start, this appeared to be successful and in Galaxy



***Step 3:*** *Visit the input dataset “HELCOM subbasins with coastal WFD waterbodies or watertypes 2022 (level 4a)”. Open the URL* *https://maps.helcom.fi/website/MADS/download/?id=67d653b1-aad1-4af4-920e-0683af3c4a48* *in a new tab and accept the data usage disclaimer to activate the download link. Then go back to the AquaINFRA platform and paste the URL* *https://maps.helcom.fi/arcgis/rest/directories/arcgisoutput/MADS/tools_GPServer/_ags_HELCOM_subbasin_with_coastal_WFD_waterbodies_or_wa.zip* *to the input field “Insert URL to a dataset” and then click on “Import to Galaxy”. This will create a .txt file in your history on Galaxy including the URL to the data.*

 I used the URL in the Upload tool in Galaxy to load this dataset too



***Step 4:*** *Click on the workflow and open it on Galaxy. Import the workflow and use the two .txt files created in Step 2 and Step 3 as inputs for the workflow.*

 It was not clear where I was supposed to click on the workflow.  I tried *Galaxy workflow:* *https://aqua.usegalaxy.eu/u/markus.konkol/w/copy-of-adj-aquainfras-daugava-workflow-shared-by-user-alabuce* but when I click on Run I get the error shown below and do not know what to do



 I also tried going to *Zenodo: Galaxy workflow.* *https://doi.org/10.5281/zenodo.15350861* . There I downloaded *Galaxy-Workflow-AquaINFRA_s_Daugava_Workflow.ga*, which I then uploaded from my local file.

**

 It appears to have been loaded, and I can Preview it.

**

 When I attempt to run it, it appears to fail at the Spatial Aggregation step.



 I do not know what is wrong and do not know where to start looking to fix it.

Is the rationale for developing the new method (or application) clearly explained?

Yes

Is the description of the method technically sound?

Partly

Are the conclusions about the method and its performance adequately supported by the findings presented in the article?

No

If any results are presented, are all the source data underlying the results available to ensure full reproducibility?

No

Are sufficient details provided to allow replication of the method development and its use by others?

No

Reviewer Expertise:

Hydrology and Hydrologic Information Systems to support data sharing

We confirm that we have read this submission and believe that we have an appropriate level of expertise to state that we do not consider it to be of an acceptable scientific standard, for reasons outlined above.

# Reviewer report: Reviewer response for version 2

**DOI:** 10.21956/openreseurope.22545.r56467

**Published:** 2025-08-08

**Authors:** Victoria Lush

**Affiliations:** 1 Aston University, Birmingham, England, UK

I would like to thank the authors for review responses. I have no further issues with the article.

Is the rationale for developing the new method (or application) clearly explained?

Yes

Is the description of the method technically sound?

Yes

Are the conclusions about the method and its performance adequately supported by the findings presented in the article?

Partly

If any results are presented, are all the source data underlying the results available to ensure full reproducibility?

Yes

Are sufficient details provided to allow replication of the method development and its use by others?

Partly

Reviewer Expertise:

Expertise include Human Computer Interaction, Data Analytics, Software Engineering, Citizen Science data, Decision Support systems.

I confirm that I have read this submission and believe that I have an appropriate level of expertise to confirm that it is of an acceptable scientific standard.

# Reviewer report: Reviewer response for version 2

**DOI:** 10.21956/openreseurope.22545.r56466

**Published:** 2025-08-08

**Authors:** Miguel Colom

**Affiliations:** 1 ENS Paris-Saclay, Université de Paris, Gif-sur-Yvette, France

In this revised version 2 of the article, the authors have given more importance to the reusability part of their work, rather than reproducibility. This is fair, since indeed from previous reviews it became clear that the proposed tool has a good potential mainly for reusability.

 A brief discussion comparison D2K with existing platforms such as Code Ocean has been added, as well as an explanation why D2K is pertinent, despite the existence of virtualization tools suck as Docker.

 Regarding PIDs, along with DOIs, now identifiers tailored specifically for software such as the SWHID are included in the discussion,  as well as the integration with Zenodo.

 In conclusion, the authors have address all my comments from the previous review, and therefore I think the article can be approved.

Is the rationale for developing the new method (or application) clearly explained?

Yes

Is the description of the method technically sound?

Partly

Are the conclusions about the method and its performance adequately supported by the findings presented in the article?

Partly

If any results are presented, are all the source data underlying the results available to ensure full reproducibility?

No source data required

Are sufficient details provided to allow replication of the method development and its use by others?

Yes

Reviewer Expertise:

Computational reproducible research

I confirm that I have read this submission and believe that I have an appropriate level of expertise to confirm that it is of an acceptable scientific standard.

# Reviewer report: Reviewer response for version 1

**DOI:** 10.21956/openreseurope.21881.r53925

**Published:** 2025-06-14

**Authors:** Victoria Lush

**Affiliations:** 1 Aston University, Birmingham, England, UK

This article presents a method designed to enable reproducible research and as such support re-use of published scientific research. The authors propose the Data-to-Knowledge Package (D2KPackage) method which packages together a collection of research materials including source code, data, virtual labs, web API services, and computational workflows. The authors also demonstrate a practical example of applying D2KPackage to a hydrological use case.

 As a whole, the article is well written and is supported with visual materials. The limitations of the proposed method are discussed.

 My main concern with the article is that the authors state that their method supports reproducibility of research, whereas D2KPackage method offers a potential solution for designing re-usable research studies. The title and some parts of the article refer to “re-usable research”, while other parts strongly suggest “reproducible research”. The authors should review their claims regarding D2KPackage support for reproducible research.

 I also do not fully agree with a discussion that reproducible research means reusable research, while usually the case, it is not guaranteed. Reusability is not the only reason for a strong push towards reproducible research, it is also about trustworthiness of the results.

 Since D2KPackage solution will not fit all research studies, it would be beneficial to clearly outline what are the prerequisites of applying this method. E.g., the authors already state that some data might not be openly available hence D2KPackage providers could crate own copy in a public repository, yet some datasets will exceed 100 files and 50GB Zenodo allowance (also duplication of data is not an ideal solution). A clear list of requirements would help potential users of D2KPackage solution to decide whether it fits their planned research study.

 Regarding the diagrams, while the authors offer a generic D2KPackage method diagram (Figure 1), it would be beneficial to include a full overview of the technologies involved to produce the D2KPackage for the hydrological case study. This would help to better appreciate what resources are typically needed to adapt a research study to fit into D2KPackage framework.

 In Figures 2 and 3 most text is cut off, the authors should use wrapped text to show content of the files.

 I am not sure if Figure 8 really adds useful visual information to this paper. This is a generic research study lifecycle diagram.

Is the rationale for developing the new method (or application) clearly explained?

Yes

Is the description of the method technically sound?

Yes

Are the conclusions about the method and its performance adequately supported by the findings presented in the article?

Partly

If any results are presented, are all the source data underlying the results available to ensure full reproducibility?

Yes

Are sufficient details provided to allow replication of the method development and its use by others?

Partly

Reviewer Expertise:

Expertise include Human Computer Interaction, Data Analytics, Software Engineering, Citizen Science data, Decision Support systems.

I confirm that I have read this submission and believe that I have an appropriate level of expertise to confirm that it is of an acceptable scientific standard, however I have significant reservations, as outlined above.

# Response

**Published:** 2025-06-30

**Authors:** Markus Konkol

We are grateful for the constructive criticism. The comments and recommendations helped us to enhance our paper considerably. In the following, we briefly outline how we addressed the raised issues. We hope that we have taken sufficient account of the comments. Don’t hesitate to request further improvements.  

 “ *My main concern with the article is that the authors state that their method supports reproducibility of research, whereas D2KPackage method offers a potential solution for designing re-usable research studies. […] The authors should review their claims regarding D2KPackage support for reproducible research.*”

**Answer:** This is indeed an important issue. For this reason, we added the section “Reproducibility” to the discussion to elaborate on this aspect.

   “ *I also do not fully agree with a discussion that reproducible research means reusable research, while usually the case, it is not guaranteed.*”

**Answer: **We fully agree with this statement and it was not our intention to convey this message. We assume this impression comes from the sentence in the introduction

 “ *A key expectation is that reproducible research results are inherently more reusable by other researchers, reducing redundant efforts and accelerating the creation of new knowledge*”? We added another sentence to clarify this: “…creation of new knowledge.****

**Nevertheless, someone trying to reuse someone else's code has to invest a lot of time to understand the code and incorporate parts of it into their own analysis**.”

   “ *Reusability is not the only reason for a strong push towards reproducible research, it is also about trustworthiness of the results.*”

**Answer: **That is correct. We added a sentence to the introduction: “Nevertheless, reproducible research should be valued not only for its intrinsic scientific merit, including verification and validation, but also for its wider benefits. **One of these advantages is that readers do not have to blindly trust the results in an article, but can check them themselves on the basis of the accompanying data and analysis.** A key expectation is that…”  

 “ *Since D2KPackage solution will not fit all research studies, it would be beneficial to clearly outline what are the prerequisites of applying this method. E.g., the authors already state that some data might not be openly available hence D2KPackage providers could crate own copy in a public repository, yet some datasets will exceed 100 files and 50GB Zenodo allowance (also duplication of data is not an ideal solution). A clear list of requirements would help potential users of D2KPackage solution to decide whether it fits their planned research study.*”

**Answer: **We added a list of *prerequisites *to the section Discussion – Use case. In the meantime, we also created a user guide with concrete steps to develop a D2K-Package as a living document, meaning that we further improve it based on user feedback. 

 “ *Regarding the diagrams, while the authors offer a generic D2KPackage method diagram (Figure 1), it would be beneficial to include a full overview of the technologies involved to produce the D2KPackage for the hydrological case study.*”

**Answer: **We added a list of all technologies used to implement the hydrological use case at the end of the chapter “Results”. We have refrained from creating another figure analogous to Figure 1, as this would have led to overloading.  

 “ *In Figures 2 and 3 most text is cut off, the authors should use wrapped text to show content of the files.*”

**Answer: **Showing the whole content of the file as wrapped text is probably too much as it contains more than 500 lines. How about adding a link to the file to the caption of the Figures 2-4?  

 “ *I am not sure if Figure 8 really adds useful visual information to this paper. This is a generic research study lifecycle diagram*.”

**Answer: **We removed the figure from the article.

# Response

**Published:** 2025-07-18

**Authors:** Markus Konkol

We are grateful for the constructive criticism. The comments and recommendations helped us to enhance our paper considerably. In the following, we briefly outline how we addressed the raised issues. We hope that we have taken sufficient account of the comments. Don’t hesitate to request further improvements.   “ *My main concern with the article is that the authors state that their method supports reproducibility of research, whereas D2KPackage method offers a potential solution for designing re-usable research studies. […] The authors should review their claims regarding D2KPackage support for reproducible research.*” **Answer:** This is indeed an important issue. For this reason, we added the section “Reproducibility” to the discussion to elaborate on this aspect.   “ *I also do not fully agree with a discussion that reproducible research means reusable research, while usually the case, it is not guaranteed.*” **Answer: **We fully agree with this statement and it was not our intention to convey this message. We assume this impression comes from the sentence in the introduction “ *A key expectation is that reproducible research results are inherently more reusable by other researchers, reducing redundant efforts and accelerating the creation of new knowledge*”? We added another sentence to clarify this: “…creation of new knowledge. **Nevertheless, someone trying to reuse someone else's code has to invest a lot of time to understand the code and incorporate parts of it into their own analysis**.”   “ *Reusability is not the only reason for a strong push towards reproducible research, it is also about trustworthiness of the results.*” **Answer: **That is correct. We added a sentence to the introduction: “Nevertheless, reproducible research should be valued not only for its intrinsic scientific merit, including verification and validation, but also for its wider benefits. **One of these advantages is that readers do not have to blindly trust the results in an article, but can check them themselves on the basis of the accompanying data and analysis.** A key expectation is that…”   “ *Since D2KPackage solution will not fit all research studies, it would be beneficial to clearly outline what are the prerequisites of applying this method. E.g., the authors already state that some data might not be openly available hence D2KPackage providers could crate own copy in a public repository, yet some datasets will exceed 100 files and 50GB Zenodo allowance (also duplication of data is not an ideal solution). A clear list of requirements would help potential users of D2KPackage solution to decide whether it fits their planned research study.*” **Answer: **We added a list of *prerequisites *to the section Discussion – Use case. In the meantime, we also created a user guide with concrete steps to develop a D2K-Package as a living document, meaning that we further improve it based on user feedback.    “ *Regarding the diagrams, while the authors offer a generic D2KPackage method diagram (Figure 1), it would be beneficial to include a full overview of the technologies involved to produce the D2KPackage for the hydrological case study.*” **Answer: **We added a list of all technologies used to implement the hydrological use case at the end of the chapter “Results”. We have refrained from creating another figure analogous to Figure 1, as this would have led to overloading.   “ *In Figures 2 and 3 most text is cut off, the authors should use wrapped text to show content of the files.*” **Answer: **Showing the whole content of the file as wrapped text is probably too much as it contains more than 500 lines. How about adding a link to the file to the caption of the Figures 2-4?   “ *I am not sure if Figure 8 really adds useful visual information to this paper. This is a generic research study lifecycle diagram*.” **Answer: **We removed the figure from the article.

# Reviewer report: Reviewer response for version 1

**DOI:** 10.21956/openreseurope.21881.r53927

**Published:** 2025-06-03

**Authors:** Miguel Colom

**Affiliations:** 1 ENS Paris-Saclay, Université de Paris, Gif-sur-Yvette, France

The submitted article proposes a the Data-to-Knowledge Package ("D2K-Package") which aims to facilitate reusability of computational methods, focusing on reproducible research. To this purpose, the package integrates several components, including virtual labs (such as MyBinder), API services, FAIR data, and workflows.

 The aim of the project is to facilitate reusability, duplication of efforts, and a easier peer review in scientific publications. A concrete use case, the hydrological dynamics of the Gulf of Riga, is presented.

 The work is clear and well written, but there are some points I think it's worth to for the authors to explain:

 In the article, reproducible research is mentioned in different parts, but however it seems that the main contribution of this article is actually reusability, as clearly highlighted in the title.

 The presented work is useful as a tool for reusability as it gathers data and code together, and allows for the creation of the virtual lab, workshops, of API services, as shown in Figure 1, but I have concerns about its potential for reproducibility. For example, it should contain automatic tests to ensure that the experiments always give the same results, or have a mandatory peer-review to ensure that the pseudocodes corresponds to the implementation, among other minimal requirements. I would, therefore, suggest the authors to relax the claims on reproducibility, since although the proposed tool has potential for it, it remains far from this objective.

 From the point of view of a reusability tool, the article lacks of comparisons with reference techniques and tools. For example: how does it compare to a Docker image which integrates an execution environment and provides its own API services? Is D2K more convenient because the "research products" in Fig. 1 would be external to the image?

 How D2K compares to a Docker image using a Jupyter notebook or any other visualization tool, with respect to using the VirtualLab? How D2K compares to existing solutions such as Code Ocean and others?

 The authors mention the use of PIDs, but a DOI is not enough for code (although the accepted solution for indexing). I'd suggest to explore other more suitable PIDs, such as the SWHID, among others.

 My conclusion is that, even if the proposed work has some potential for a reusability work, the reasons why it would facilitate reproducibility are not well justified in this version of the article. Given that it also lacks a proper comparison with other alternatives to reuse code, I think it needs to be completed following these recommendations, before being indexed as a method article, or to be proposed as a general solution to the community. I strongly recommend the authors to improve this word and prepare a new revision.

Is the rationale for developing the new method (or application) clearly explained?

Yes

Is the description of the method technically sound?

Partly

Are the conclusions about the method and its performance adequately supported by the findings presented in the article?

Partly

If any results are presented, are all the source data underlying the results available to ensure full reproducibility?

No source data required

Are sufficient details provided to allow replication of the method development and its use by others?

Yes

Reviewer Expertise:

Computation reproducible research

I confirm that I have read this submission and believe that I have an appropriate level of expertise to confirm that it is of an acceptable scientific standard, however I have significant reservations, as outlined above.

# Response

**Published:** 2025-06-30

**Authors:** Markus Konkol

We are grateful for the constructive criticism. The comments and recommendations helped us to enhance our paper considerably. In the following, we briefly outline how we addressed the raised issues. We hope that we have taken sufficient account of the comments. Don’t hesitate to request further improvements.   “ *I have concerns about its potential for reproducibility. For example, it should contain automatic tests to ensure that the experiments always give the same results, or have a mandatory peer-review to ensure that the pseudocodes correspond to the implementation, among other minimal requirements. I would, therefore, suggest the authors to relax the claims on reproducibility, since although the proposed tool has potential for it, it remains far from this objective.*”

**Answer: **We fully agree with this comment and have therefore added a further section “Reproducibility” to the discussion, in which this aspect is highlighted.  

 “ *From the point of view of a reusability tool, the article lacks of comparisons with reference techniques and tools. For example: how does it compare to a Docker image which integrates an execution environment and provides its own API services? Is D2K more convenient because the "research products" in Fig. 1 would be external to the image? How D2K compares to a Docker image using a Jupyter notebook or any other visualization tool, with respect to using the VirtualLab? How D2K compares to existing solutions such as Code Ocean and others?*”

**Answer:** This is indeed a crucial gap. We have therefore added a corresponding section at the beginning of the discussion.  

 “ *The authors mention the use of PIDs, but a DOI is not enough for code (although the accepted solution for indexing). I'd suggest to explore other more suitable PIDs, such as the SWHID, among others.*”

**Answer: **During our research on PIDs for source code and SWHID we found out that fortunately there is already an integration between Zenodo and Software Heritage. We have explained this aspect in more detail in the section “Persistence“.

# Response

**Published:** 2025-07-18

**Authors:** Markus Konkol

We are grateful for the constructive criticism. The comments and recommendations helped us to enhance our paper considerably. In the following, we briefly outline how we addressed the raised issues. We hope that we have taken sufficient account of the comments. Don’t hesitate to request further improvements.   “ *I have concerns about its potential for reproducibility. For example, it should contain automatic tests to ensure that the experiments always give the same results, or have a mandatory peer-review to ensure that the pseudocodes correspond to the implementation, among other minimal requirements. I would, therefore, suggest the authors to relax the claims on reproducibility, since although the proposed tool has potential for it, it remains far from this objective.*” **Answer: **We fully agree with this comment and have therefore added a further section “Reproducibility” to the discussion, in which this aspect is highlighted.   “ *From the point of view of a reusability tool, the article lacks of comparisons with reference techniques and tools. For example: how does it compare to a Docker image which integrates an execution environment and provides its own API services? Is D2K more convenient because the "research products" in Fig. 1 would be external to the image? How D2K compares to a Docker image using a Jupyter notebook or any other visualization tool, with respect to using the VirtualLab? How D2K compares to existing solutions such as Code Ocean and others?*” **Answer:** This is indeed a crucial gap. We have therefore added a corresponding section at the beginning of the discussion.   “ *The authors mention the use of PIDs, but a DOI is not enough for code (although the accepted solution for indexing). I'd suggest to explore other more suitable PIDs, such as the SWHID, among others.*” **Answer: **During our research on PIDs for source code and SWHID we found out that fortunately there is already an integration between Zenodo and Software Heritage. We have explained this aspect in more detail in the section “Persistence“.
